# Supplementary material for: Deciphering changes in the incidence of hemorrhagic stroke and cerebral venous sinus thrombosis during the coronavirus disease 2019 pandemic: A nationwide time-series correlation study
Source: PLoS One. 2024 Oct 3;19(10):e0301313. doi: 10.1371/journal.pone.0301313 (PMC11449313; doi:10.1371/journal.pone.0301313)
Supplement: S1 File — (DOCX) [file pone.0301313.s001.docx]

**SUPPLEMENTARY MATERIAL**

**Supplementary material 1. Visualization of weighted ranks as known risk factors hemorrhagic stroke or CVST from 2007 to 2022 through bump charts.** Literature-based weights were as follows: antiplatelet therapy, 4.96; oral anticoagulant therapy, 4.8; hypertension, 2.55; atrial fibrillation, 1.92; chronic liver disease, 2.67; end-stage liver disease, 4.18; intracranial aneurysm, 3.20; hormonal agents, 7.59; cancer, 4.86; and severe infection, 4.5. References are available in the main text. Data on arteriovenous malformations, Marfan syndrome, Moyamoya disease, and autosomal dominant polycystic kidney disease were not used in the analysis as the reference studies were small in size or odds ratios were not generated compared to the general population. (a) I60+I61. (c) I67.6.

**
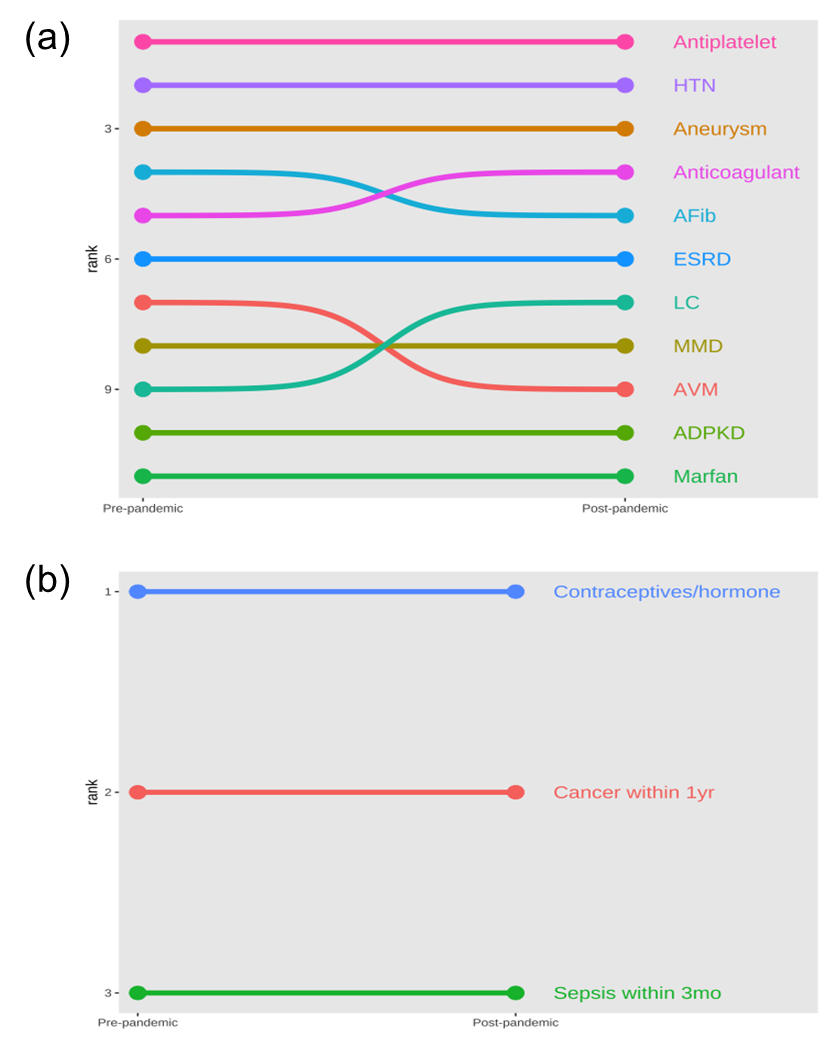
**

Abbreviations: HTN, hypertension; ESRD, end-stage renal disease; Afib, Atrial fibrillation; LC, liver cirrhosis; ADPKD, autosomal dominant polycystic kidney disease; MMD, Moyamoya disease; AVM, arteriovenous malformation.

**Supplementary material 2. Observed and predicted incidence of I60, I61, and I67.6 spread by months during the pandemic period (2020–2022).**

| **Time** | **I60 obs.** | **I60 pred.** | **CI95L** | **CI95U** | **RR** | **I61 obs.** | **I61 pred.** | **CI95L** | **CI95U** | **RR** | **I67.6 obs.** | **I67.6 pred.** | **CI95L** | **CI95U** | **RR** |
| --- | --- | --- | --- | --- | --- | --- | --- | --- | --- | --- | --- | --- | --- | --- | --- |
| 2020-01-01 | 0.61 | 0.62 | 0.57 | 0.68 | 0.98 | 1.41 | 1.42 | 1.29 | 1.55 | 0.99 | 0.009 | 0.008 | 0.004 | 0.012 | 1.20 |
| 2020-02-01 | 0.57 | 0.58 | 0.52 | 0.63 | 0.99 | 1.28 | 1.31 | 1.18 | 1.45 | 0.98 | 0.010 | 0.008 | 0.004 | 0.012 | 1.36 |
| 2020-03-01 | **0.52** | **0.63** | **0.58** | **0.69** | **0.83** | **1.24** | **1.41** | **1.28** | **1.54** | **0.88** | 0.009 | 0.007 | 0.003 | 0.011 | 1.30 |
| 2020-04-01 | 0.60 | 0.61 | 0.56 | 0.67 | 0.98 | 1.28 | 1.34 | 1.21 | 1.48 | 0.95 | 0.010 | 0.007 | 0.003 | 0.011 | 1.45 |
| 2020-05-01 | 0.59 | 0.62 | 0.57 | 0.68 | 0.95 | 1.21 | 1.29 | 1.15 | 1.42 | 0.94 | 0.008 | 0.008 | 0.004 | 0.012 | 0.92 |
| 2020-06-01 | 0.56 | 0.57 | 0.51 | 0.63 | 0.99 | 1.09 | 1.13 | 1.00 | 1.27 | 0.96 | **0.004** | **0.008** | **0.004** | **0.012** | **0.47** |
| 2020-07-01 | 0.53 | 0.55 | 0.49 | 0.61 | 0.96 | 1.15 | 1.05 | 0.92 | 1.19 | 1.09 | 0.008 | 0.008 | 0.004 | 0.012 | 0.92 |
| 2020-08-01 | 0.57 | 0.56 | 0.50 | 0.61 | 1.02 | 0.98 | 1.04 | 0.90 | 1.18 | 0.94 | 0.006 | 0.008 | 0.004 | 0.012 | 0.72 |
| 2020-09-01 | 0.55 | 0.58 | 0.52 | 0.64 | 0.95 | 1.20 | 1.14 | 1.00 | 1.28 | 1.05 | 0.009 | 0.007 | 0.002 | 0.011 | 1.45 |
| 2020-10-01 | **0.57** | **0.65** | **0.59** | **0.70** | **0.88** | 1.31 | 1.30 | 1.17 | 1.44 | 1.00 | 0.007 | 0.008 | 0.004 | 0.012 | 0.81 |
| 2020-11-01 | 0.59 | 0.63 | 0.57 | 0.69 | 0.94 | 1.29 | 1.37 | 1.23 | 1.51 | 0.94 | 0.009 | 0.006 | 0.002 | 0.010 | 1.46 |
| 2020-12-01 | **0.57** | **0.65** | **0.59** | **0.71** | **0.88** | 1.35 | 1.40 | 1.26 | 1.54 | 0.96 | 0.005 | 0.007 | 0.003 | 0.011 | 0.67 |
| 2021-01-01 | 0.58 | 0.62 | 0.56 | 0.68 | 0.94 | 1.39 | 1.40 | 1.26 | 1.55 | 0.99 | 0.006 | 0.008 | 0.004 | 0.013 | 0.68 |
| 2021-02-01 | 0.55 | 0.58 | 0.52 | 0.64 | 0.94 | 1.28 | 1.29 | 1.15 | 1.44 | 0.99 | 0.005 | 0.008 | 0.004 | 0.013 | 0.58 |
| 2021-03-01 | 0.60 | 0.64 | 0.58 | 0.70 | 0.94 | 1.31 | 1.39 | 1.24 | 1.54 | 0.94 | 0.009 | 0.008 | 0.003 | 0.012 | 1.21 |
| 2021-04-01 | 0.56 | 0.61 | 0.55 | 0.67 | 0.92 | 1.36 | 1.32 | 1.17 | 1.47 | 1.03 | 0.009 | 0.008 | 0.003 | 0.012 | 1.11 |
| 2021-05-01 | 0.59 | 0.62 | 0.56 | 0.68 | 0.94 | 1.30 | 1.26 | 1.11 | 1.41 | 1.03 | **0.019** | **0.009** | **0.004** | **0.013** | **2.16** |
| 2021-06-01 | 0.55 | 0.57 | 0.51 | 0.63 | 0.96 | 1.04 | 1.11 | 0.96 | 1.26 | 0.94 | **0.016** | **0.009** | **0.004** | **0.013** | **1.87** |
| 2021-07-01 | 0.50 | 0.55 | 0.49 | 0.61 | 0.91 | 1.01 | 1.03 | 0.87 | 1.18 | 0.99 | **0.016** | **0.009** | **0.004** | **0.013** | **1.84** |
| 2021-08-01 | **0.49** | **0.56** | **0.50** | **0.62** | **0.88** | 1.06 | 1.01 | 0.86 | 1.17 | 1.05 | 0.007 | 0.008 | 0.004 | 0.013 | 0.79 |
| 2021-09-01 | 0.53 | 0.58 | 0.52 | 0.65 | 0.91 | 1.07 | 1.11 | 0.96 | 1.27 | 0.97 | 0.011 | 0.007 | 0.002 | 0.012 | 1.61 |
| 2021-10-01 | **0.56** | **0.65** | **0.58** | **0.71** | **0.87** | 1.24 | 1.27 | 1.12 | 1.43 | 0.97 | **0.016** | **0.009** | **0.004** | **0.014** | **1.85** |
| 2021-11-01 | **0.56** | **0.63** | **0.56** | **0.70** | **0.88** | 1.29 | 1.34 | 1.18 | 1.50 | 0.96 | 0.008 | 0.006 | 0.001 | 0.011 | 1.19 |
| 2021-12-01 | **0.56** | **0.65** | **0.58** | **0.72** | **0.86** | 1.30 | 1.37 | 1.21 | 1.53 | 0.95 | 0.010 | 0.008 | 0.003 | 0.013 | 1.38 |
| 2022-01-01 | **0.52** | **0.62** | **0.56** | **0.69** | **0.83** | 1.30 | 1.37 | 1.20 | 1.54 | 0.95 | 0.009 | 0.009 | 0.004 | 0.014 | 1.06 |
| 2022-02-01 | **0.48** | **0.58** | **0.51** | **0.65** | **0.83** | 1.18 | 1.26 | 1.09 | 1.43 | 0.94 | 0.008 | 0.009 | 0.003 | 0.014 | 0.87 |
| 2022-03-01 | **0.51** | **0.64** | **0.56** | **0.71** | **0.80** | **1.14** | **1.35** | **1.18** | **1.52** | **0.84** | **0.003** | **0.008** | **0.003** | **0.014** | **0.34** |
| 2022-04-01 | **0.52** | **0.61** | **0.54** | **0.68** | **0.84** | 1.15 | 1.28 | 1.11 | 1.46 | 0.90 | 0.009 | 0.008 | 0.003 | 0.014 | 1.04 |
| 2022-05-01 | 0.56 | 0.62 | 0.55 | 0.70 | 0.90 | 1.21 | 1.22 | 1.04 | 1.40 | 0.99 | 0.011 | 0.009 | 0.004 | 0.015 | 1.23 |
| 2022-06-01 | 0.55 | 0.57 | 0.50 | 0.64 | 0.97 | 1.05 | 1.06 | 0.88 | 1.24 | 0.99 | **0.015** | **0.009** | **0.004** | **0.015** | **1.66** |
| 2022-07-01 | **0.46** | **0.55** | **0.47** | **0.63** | **0.83** | 1.03 | 0.98 | 0.80 | 1.16 | 1.05 | 0.008 | 0.009 | 0.004 | 0.015 | 0.82 |

Abbreviations: obs, observed; pred, predicted; C95L, lower limit of 95% confidence interval; C95U, upper limit of 95% confidence interval; RR, rate ratio.

**Supplementary material 3. Prevalence of known risk factor in incident hemorrhagic stroke and CVST cases; pre-pandemic (2007–2019) vs post-pandemic (2020–2022).**

|  | | **2007–2019 prevalence** | **2020–2022 prevalence** | **Pre-pandemic**  **vs pandemic** |
| --- | --- | --- | --- | --- |
| I60 | Antiplatelet therapy | 0.136 | 0.223 | 0.087(0.081–0.093) |
|  | Anticoagulant therapy | 0.013 | 0.026 | 0.013(0.01–0.015) |
|  | Hypertension | 0.048 | 0.073 | 0.025(0.021–0.029) |
|  | End stage renal disease | 0.005 | 0.010 | 0.005(0.004–0.007) |
|  | Atrial fibrillation | 0.018 | 0.028 | 0.01(0.007–0.012) |
|  | Liver cirrhosis | 0.005 | 0.013 | 0.008(0.006–0.01) |
|  | Marfan syndrome | 0.000 | 0.000 | 0(0–0) |
|  | ADPKD | 0.001 | 0.003 | 0.002(0.001–0.003) |
|  | Moyamoya disease | 0.004 | 0.006 | 0.002(0–0.003) |
|  | Intracranial aneurysm | 0.120 | 0.137 | 0.017(0.012–0.022) |
|  | Arteriovenous malformation | 0.007 | 0.008 | 0.001(−0.001–0.002) |
| I61 | Antiplatelet therapy | 0.228 | 0.359 | 0.131(0.126–0.136) |
|  | Anticoagulant therapy | 0.041 | 0.072 | 0.031(0.028–0.033) |
|  | Hypertension | 0.074 | 0.107 | 0.033(0.029–0.036) |
|  | End stage renal disease | 0.017 | 0.032 | 0.015(0.013–0.017) |
|  | Atrial fibrillation | 0.044 | 0.069 | 0.026(0.023–0.028) |
|  | Liver cirrhosis | 0.011 | 0.026 | 0.015(0.014–0.017) |
|  | Marfan syndrome | 0.000 | 0.000 | 0(0–0) |
|  | ADPKD | 0.001 | 0.002 | 0.001(0–0.001) |
|  | Moyamoya disease | 0.015 | 0.022 | 0.007(0.006–0.009) |
|  | Intracranial aneurysm | 0.031 | 0.054 | 0.023(0.021–0.025) |
|  | Arteriovenous malformation | 0.014 | 0.012 | −0.001(−0.003–0) |
| I67.6 | Contraceptives and hormones ≤ 90 d | 0.030 | 0.036 | 0.007(−0.017–0.034) |
|  | Delivery ≤ 90 d | 0.000 | 0.000 | 0(−0.005–0.009) |
|  | Sepsis within ≤ 90 d | 0.002 | 0.000 | −0.002(−0.008–0.008) |
|  | Trauma within ≤ 90 d | 0.000 | 0.000 | 0(−0.005–0.009) |
|  | Cancer within ≤ 1 y | 0.039 | 0.033 | −0.006(−0.03–0.021) |

Abbreviation: ADPKD, autosomal dominant polycystic kidney disease.
